# 

**Published:** 1907-02-23

**Authors:** 


### BOOKS RECEIVED.

Baillière, Tindall, and Cox.

"The Prevention of Cancer." By C. B. Keetley, F.R.C.S.

W. B. Clive.

"Matriculation Directory." No. XLV. January 1907.

### Special Text Books for Nurses.

### NURSING (General).

**A HANDBOOK FOR NURSES.** 5/- net. 5/4 Post free.

By J. K. WATSON, M.D., M.B., C.M. New and Revised Edition. Profusely Illustrated.

**NURSING: ITS THEORY AND PRACTICE.** 3/6 Post free.
Being a complete Text-book of Medical, Surgical, and Monthly Nursing. By PERCY G. LEWIS, M.D., M R.C.S., L.S.A. New Edition, Enlarged and Revised. Profusely Illustrated.

**NURSING: ITS PRINCIPLES AND PRACTICE.** 7/6 net. 7/10 Post free.

By ISABEL ADAMS HAMPTON. Third Edition, Revised and Enlarged. Illustrated.

### FEVER NURSING.

**LECTURES UPON THE NURSING OF INFECTIOUS DISEASES.** 2/6 net. 2/9 Post free.

By F. J. WOOLLACOTT, M.A., M.D.

**FEVERS AND INFECTIOUS DISEASES:** 1/- Post free.
Their Nursing and Practical Management. By WILLIAM HARDING, M.D. Ed., M.R.C.P.

**NURSING OF TROPICAL FEVERS.** 1/6 net. 1/8 Post free.

By S. F. POLLARD, late Sister, Army Nursing Reserve.

### MENTAL NURSING.

**MENTAL NURSING.** 1/- Post free.

By WILLIAM HARDING, M.B. Ed., M.R.C.P. Lond.

**THE CARE AND NURSING OF THE INSANE.** 1/- net. 1/2 Post free.

By PERCY J. BAILY, M.B., C.M. Edin.

Part I. ANATOMY AND PHYSIOLOGY. Illustrated.

**OUTLINES OF INSANITY.** 1/6 Post free.

A Popular Treatise on the Salient Features of Insanity. By FRANCIS H. WALMSLEY, M.D. Popular Edition, stitched.

### SURGICAL NURSING.

**ABDOMINAL SURGERY.** 2/6 net. 2/9 Post free.

A Manual for Nurses. By HAROLD BURROWS, M.B., F.R.C.S. Illustrated.

**SURGICAL INSTRUMENTS AND APPLIANCES USED IN OPERATIONS.** 1/6 net. 1/8 Post free.
An Illustrated and Classified List, with Explanatory Notes. By HAROLD BURROWS, M.B.Lond., B.S., F.R.C.S. New Edition, Revised, with special New Chapter on Sterilisation of Instruments.

**SURGICAL WARD-WORK AND NURSING.** 3/6 net. 3/10 Post free.

By ALEXANDER MILES, M.D. Edin., C.M., F.R.C.S.E. Second Edition, Enlarged and entirely Revised. Copiously Illustrated.

### OBSTETRIC NURSING.

**A COMPLETE HANDBOOK OF MIDWIFERY.** 6/- net. 6/4 Post free.
By J. K. WATSON, M.D.
**Especially designed to meet the requirements of Candidates for the C.M.B. Examinations, and is undoubtedly the best and most up-to-date text-book, and its use will do much to ensure success. Profusely Illustrated.**

**HOW TO BECOME A CERTIFIED MIDWIFE.** 1/- net. 1/1 Post free.

By E. L. C. APPEL, M.B., B.S., B.Sc. Lond. Crown 8vo. bound limp cloth.

**SIMPLE INTRODUCTORY LESSONS ON MIDWIFERY.** 1/- net. 1/1 Post free.

By M. LOANE. Illustrated.

**THE MIDWIVES' POCKET BOOK** 1/- Post free.

By HONNOR MORTEN.

**INSTRUCTIONS TO MIDWIVES.** 6d. net. 7d. Post free.

Compiled by the Midwives' Supervising Committee of the Corporation of Manchester.

**The Scientific Press, Ltd.,**

**Publishers of Nursing Manuals, Text Books, and Charts of all descriptions.**

*COMPLETE CATALOGUE ON APPLICATION.*

**28 & 29 SOUTHAMPTON STREET, STRAND, LONDON. W.C.**

